# Retrospective review of 27 European cases of fatal elephant endotheliotropic herpesvirus-haemorrhagic disease reveals evidence of disseminated intravascular coagulation

**DOI:** 10.1038/s41598-021-93478-0

**Published:** 2021-07-08

**Authors:** K. L. Perrin, A. T. Kristensen, M. F. Bertelsen, D. Denk

**Affiliations:** 1 Center for Zoo and Wild Animal Health, Copenhagen Zoo, Roskildevej 38, 2000 Frederiksberg, Denmark; 2 The Department of Veterinary Clinical Sciences, Faculty of Health and Medical Sciences, University of Copenhagen, Dyrlægevej 16, 1870 Frederiksberg, Denmark; 3 International Zoo Veterinary Group, Station House, Parkwood Street, Keighley, BD21 4NQ UK; 4 Present Address: Institute for Animal Pathology, Ludwig-Maximilians-University, Veterinärstr. 13, 80539 Munich, Germany

**Keywords:** Viral infection, Vascular diseases

## Abstract

Elephant endotheliotropic herpesvirus haemorrhagic disease (EEHV-HD) is widely acknowledged as the most common cause of mortality in young Asian elephants (*Elephas maximus*) in captivity. The objective of the current study was to perform a blinded, retrospective pathology review of European EEHV-HD fatalities, constituting the largest systematic assessment of EEHV-HD pathology to date. Findings between viral genotypes were compared with the aim to investigate if disseminated intravascular coagulation (DIC) could be substantiated as a significant complicating factor, thereby increasing the understanding of disease pathophysiology. Immunohistochemical staining confirmed endothelial cell (EC) damage and the presence of EC intranuclear inclusion bodies, demonstrating a direct viral cytopathic effect. Microthrombi were observed in 63% of cases in several organs, including lungs, which, together with widespread haemorrhage and thrombocytopenia reported in EEHV-HD case reports, supports the presence of overt DIC as a serious haemostatic complication of active EEHV infection. Death was attributed to widespread vascular damage with multi-organ dysfunction, including severe acute myocardial haemorrhage and subsequent cardiac failure. Systemic inflammation observed in the absence of bacterial infection may be caused by cytokine release syndrome. Findings reinforce the necessity to investigate cytokine responses and haemostatic status during symptomatic and asymptomatic EEHV viraemia, to potentially support the use of anti-inflammatory treatment in conjunction with anti-viral therapy and cardiovascular support.

## Introduction

Elephant endotheliotropic herpesvirus (EEHV) infection is reported to be the most common cause of mortality in young Asian elephants (*Elephas maximus*) in Europe and North America^1,2^. EEHV infection is believed to be endemic in adult populations^3^ and species-specific genotypes have co-evolved alongside their host elephant species^1^. Despite this, fatal infection is associated with acquired thrombocytopenia, monocytopenia and haemorrhagic diathesis in juveniles^4–7^. While a strict case definition is yet to be agreed upon, clinical disease is called EEHV-haemorrhagic disease (EEHV-HD), to distinguish between viraemic elephants with clinical signs, and those that are viraemic but asymptomatic. Rapidly increasing viraemia is associated with decreasing platelet counts, heterophilia/monocytopenia and onset of clinical signs^4–6,8,9^. The first case of EEHV was reported in a Swiss elephant in 1990^10,11^ and multiple case reports in captive and wild elephants have been presented in the peer-reviewed literature since then^12–19^. There are seven known genotypes (EEHV-1 to -7) and while the majority of fatal Asian elephant cases are associated with EEHV-1^2,11,20–23^, differences in lesion distribution have been suggested based on case reports of EEHV-HD caused by EEHV-3^12^, EEHV-4^15,19^ and EEHV-5^14,24^. EEHVs are in the family *Proboscivirus* and are most closely related to *Betaherpesvirinae*. However they are phylogenetically distant enough that a new Herpesvirus family has been proposed^25^.

Acquired thrombocytopenia is a consistently reported feature of EEHV-HD cases^4,6,8,9^. In humans acquired thrombocytopenia is most commonly caused by disseminated intravascular coagulation (DIC)^26^. DIC is defined as “*an acquired syndrome characterised by the intravascular activation of coagulation with loss of localisation arising from different causes. It can originate from and cause damage to the microvasculature, which if sufficiently severe, can produce organ dysfunction*”^27^. Balanced haemostasis relies on localised reactions to coagulate blood in response to blood vessel injury, and rapid breakdown of the clot (fibrinolysis) when no longer needed. Damage to endothelial cells exposes circulating platelets to extra-luminal tissue factor, which is the primary stimulus for coagulation^28^. Tissue factor exposure can activate platelets, and activated inflammatory cells, such as monocytes, can further express tissue factor, resulting in a procoagulant state^28–31^. When homeostatic mechanisms become overwhelmed, generalised activation of coagulation distant to the site of endothelial injury occurs, leading to compensated or non-overt DIC^27^. If the underlying cause is not resolved, consumption of platelets and clotting factors may result in a decompensated state, known as overt DIC, which is characterised by the combination of microthrombi and bleeding^27^. Increased break-down of thrombi (hyperfibrinolysis) may exacerbate consumption of platelets and clotting factors, leading to further clinical bleeding^32^. DIC is diagnosed in the living patient by using scoring systems to assess thrombocytopenia as well as indirect measures of hypocoagulability e.g. plasma clotting times, and thrombosis, e.g. fibrin degradation products or D-dimer^27,33^. Currently it is unclear whether EEHV-HD is associated with DIC. Although thrombosis has been reported in two cases^12^, systematic examination of large numbers of cases has not been performed to date. The authors hypothesise that EEHV-HD is associated with both thrombosis and haemorrhage and this, in light of reports that thrombocytopenia is a consistent feature of EEHV-HD^4,6,8,9^, fulfils the criteria for diagnosis of overt DIC.

The objective of this retrospective investigation was to describe clinical information and postmortem lesions observed across all known European EEHV-HD fatalities between 1985 and 2017, and to compare findings between different viral genotypes with the overall aim to investigate if DIC is a significant complicating factor, thereby increasing the understanding of the pathophysiology of EEHV infection in Asian elephants.

## Methods

### EEHV case inclusion and exclusion criteria

The study protocol was approved by the Institutional Animal Care and Use Committee at Copenhagen Zoo (Frederiksberg, Denmark), as well as individually by contributing institutions. Fatal cases of EEHV-HD in the European population of Asian elephants were identified from published reports and discussion with the elephant Taxon Advisory Group veterinary advisors, as well as zoo veterinarians and pathologists. EEHV-HD fatalities were defined as Asian elephant deaths with positive EEHV-specific polymerase chain reaction (PCR) analysis on whole blood or other tissues, and compatible macroscopic lesions including widespread haemorrhage and oedema. Pathology reports and formalin fixed materials were requested from holding institutions, and additional case information was obtained from published literature and conference proceedings. Cases were excluded if no formalin-fixed tissue was available. EEHV PCR-negative control tissues were identified from archived cases at the International Zoo Veterinary Group (Keighley, UK) and Copenhagen Zoo (Frederiksberg, Denmark).

### Histopathology and immunohistochemistry

Tissues submitted in 10% formalin were processed routinely and embedded in paraffin wax. Paraffin blocks were sectioned and the slides stained with haematoxylin and eosin (HE) following standard histology operating procedures. Slides were scanned with a 20 × objective lens and 2 × optical magnification changer (lens 20x/0.75 NA Plan Apo scanned with Leica Aperio AT2 brightfield digital pathology scanner, Leica Biosystems Division of Leica Microsytems Inc., Illinois, USA). Scanned slides were examined using CaseViewer 2.3 (3DHISTECH Ltd., Budapest, Hungary) or Aperio ImageScope 12.4 (Leica Biosystems Division of Leica Microsystems Inc.).

In addition, selected slides were stained with Martius, Scarlet and Blue (MSB), Perls’ Prussian blue or Ziehl–Neelsen methods. Von Willebrand factor immunohistochemistry using routine polymer detection methods was performed to visualise endothelial cells of selected tissues and cases. Briefly, samples were routinely deparaffinised, rehydrated (PT Link, Agilent Dako, Santa Clara, California, USA) and heat-mediated epitope retrieval was performed (code No. K8005, EnVision FLEX Target Retrieval System Low pH, Dako Denmark ApS, Glostrup, Denmark). An autostainer (Autostainer Link 48, Agilent Dako) was then used to apply an endogenous enzyme blocker (code No. SM801, EnVision FLEX Peroxidase-blocking reagent, Dako Denmark ApS), prior to incubation with polyclonal rabbit anti-human von Willebrand factor antibody (code No. A0082, diluted 1:2000 in code No. K8006, EnVision FLEX antibody diluent, Dako Denmark ApS) for 20 min. A labelled polymer (code No. K4003, EnVision + System-HRP Labelled Polymer α rabbit, Dako Denmark ApS) and the antigen-primary antibody visualisation (code No. SM802, EnVision FLEX/HRP, Dako Denmark ApS) were applied. Lastly, a substrate chromogen (code No. DM827, EnVision FLEX DAB + Chromogen and code No. SM802, EnVision Substrate Buffer, Dako Denmark ApS) was applied, followed by an HE counter-stain. All methods were carried out in accordance with relevant guidelines and regulations and appropriate control tissue.

All tissues available from each case were examined independently by two blinded evaluators, one of which was a European College of Veterinary Pathology board certified pathologist. All tissues were scored for oedema, haemorrhage and inflammation as either absent, mild, moderate or severe and scores were recorded in Microsoft Excel (Microsoft Office Professional Plus 2016, Microsoft Corporation, Washington, USA). Congestion was scored in liver, spleen, kidney and lung; lymphoid depletion in spleen, lymph nodes and thymus. Erythrophagocytosis was scored in lymph nodes and spleen. Where the lesion degree varied across tissue sections, the highest score evident was recorded. Obtained results were then compared between evaluators and, where different, a consensus score was agreed upon based on joint slide review. Intranuclear inclusion bodies (INIB) were quantified as the average number observed in 10 high power fields (× 400; HPF) for all tissues. Scores were assigned as none observed (no INIB seen during assessment of HPF, or during screening of tissues), rare (average of less than 1 INIB per 10 HPF), moderate (average of 1–4 INIB per 10 HPF) and frequent (> 4 INIB seen per 10 HPF). Vascular changes including vessel wall oedema, endothelial cell damage and leukocyte migration evident in liver and lung and were scored as present or absent. Lung and selected renal sections were stained with MSB to identify fibrin.

To analyse the association between lesion scores and duration of clinical signs prior to death, scoring data were ordered by duration of clinical signs and visually examined for trends. Where a potential association existed, Spearman correlation was performed using GraphPad Prism (GraphPad Software, San Diego, California, USA) to assess the association between duration of clinical signs and lesion severity scores^34^. Significance was set at *p* < 0.05.

## Results

### Cases

A total of 31 fatalities caused by EEHV-HD were retrospectively identified from 17 institutions in Europe between 1985 and 2018^2,13^. Tissues were available from 27 cases (87%), of which 12 were females and 15 males. Twenty-three deaths were caused by EEHV genotype 1A, and there were one case each of EEHV-1B (7.6 years, female), EEHV-5 (1.7 years, male)^14,24^ and a co-infection of EEHV-1A and -4 (1.6 years, male). Tissue viral loads for this co-infection indicated that EEHV-4 was the cause of death^15^. One additional EEHV-1 case did not have nucleotide sequencing information to allow identification of the virus subtype. Findings in this case were similar to those of the EEHV-1A cases and are therefore presented together. Median age of death of the included cases was 984 days (2.7 years) and ranged from 392 to 2765 days old (1.1–7.6 years). Body weight ranged from 452 to 2000 kg (median 963 kg).

### Clinical presentation and paraclinical findings

Case histories were obtained from published information and personal communication with clinicians for 24 cases (89%)^4,10,11,13–15,24,35^. The duration of clinical signs prior to death (spontaneous or euthanasia) varied from 0 to 7 days; zero (n = 1), one (n = 5), two (n = 5 and the EEHV-1B case), three (n = 4 and the EEHV-1A/4 case), four (n = 4), five (n = 2), six (n = 2 and the EEHV-5 case) or seven (n = 1) days. One case had a low viraemia associated with mild lethargy 34 days prior to death. Subsequent samples were EEHV-PCR negative until 10 days prior to death, at which time this individual was being treated for a temporal gland abscess^4^. Viraemia was detected with whole blood PCR prior to clinical signs in two cases, and prior to death in a further six cases. Only five cases had both antemortem PCR and haemogram results available. Platelet counts were available for seven cases and ranged from 13 to 133 × 10^9^/L (median 55 × 10^9^/L). All were considered thrombocytopenic based on individual values (where available) or population-derived reference values^36,37^.

The amount of clinical information varied widely across cases. Lethargy (16/24) and/or non-specific illness/depression (16/24) were the most common clinical signs, followed by hyporexia or anorexia (12/24) and gastrointestinal signs including colic (n = 5), bloating (n = 1), increased flatulence (n = 1), dry (n = 3) or soft faeces (n = 2) or reduced frequency of defecation (n = 1). Musculoskeletal signs were recorded for three calves which included stiffness, lameness and a swollen joint. Additional recorded clinical signs included polydipsia, anuria, restlessness, temporal gland infection, hanging of the trunk and slow recovery from sedation or anaesthesia. Information about body temperature was recorded for 13 elephants and considered elevated (pyrexia) for ten. Oral ulceration was noted in two cases and discolouration in five cases. The tongue was noted to be pale (n = 1), discoloured (n = 1), purple or blue (n = 5) and/or swollen (n = 4).

Treatment was attempted in 20 (74%) cases, no treatment was given in five (19%) cases, and records were not available for two (7%) cases. Treatments given were as follows; antiviral (n = 14, 52%; famciclovir and/or ganciclovir or acyclovir), broad spectrum antibiotics (n = 11, 41%), non-steroidal anti-inflammatories (n = 11, 41%), fluid therapy (n = 8, 30%; intravenous and/or rectal), elephant plasma (n = 5, 19%), glucocorticoids (n = 5, 19%), hyoscine (n = 4, 15%), methadone (n = 2, 7%), elephant whole blood transfusion and recombinant factor VIIa (n = 1, 4%), oral paraffin (n = 1, 4%), furosemide and interferon (n = 1, 4%). Eight cases received an EEHV-specific treatment protocol including anti-viral medication, fluids and elephant plasma administration, but for three of these cases the protocol was initiated on the day of death. Elephants receiving this protocol tended to survive for longer (2–7 days) compared to those receiving no treatment (0–1 day). Elephants given symptomatic treatment such as antibiotic and/or non-steroidal anti-inflammatory therapy survived for 1–5 days. Where antiviral treatment without fluid therapy or plasma was administered the survival time was 2–5 days. Death was spontaneous in 23 (85%) cases, due to euthanasia in three (11%) cases, and not reported in one case.

### Gross postmortem findings

**Figure 1:**
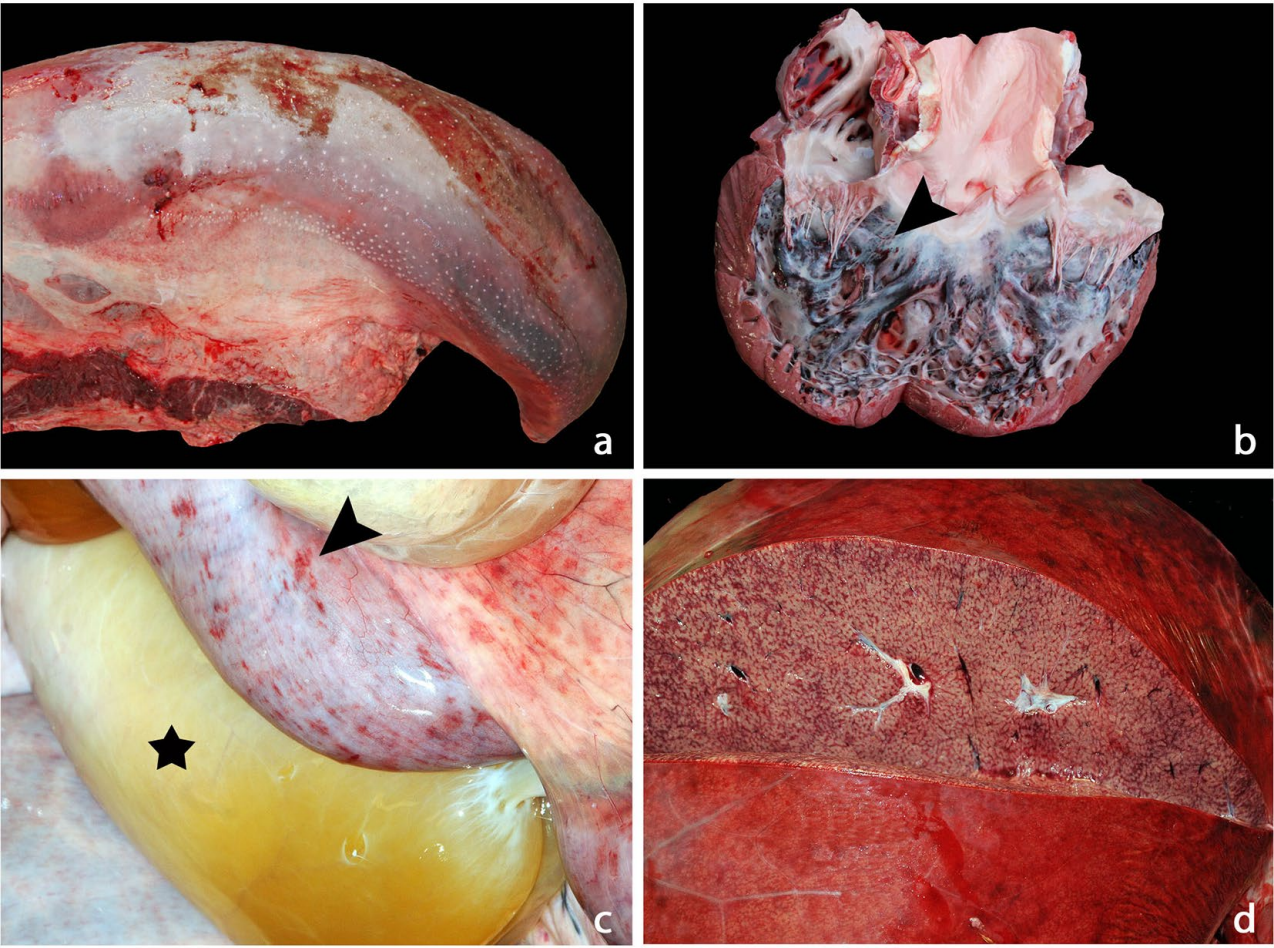
Representative gross lesions from Asian elephant endotheliotropic herpesvirus-haemorrhagic disease fatalities. (**a**) Case 5. Tongue after pluck removal. There is diffuse lingual swelling and cyanosis. (**b**) Case 5. Heart, sectioned. Severe subendocardial petechial (arrow head), ecchymosal and suffusive haemorrhages are present. (**c**) Case 26. Abdominal cavity, view of intestines and mesentery. The small intestine presents with multifocal petechial to ecchymosal serosal and subserosal haemorrhages (arrow head). Extensive oedema expanding the mesentery (star). Image courtesy of Jonathan Cracknell. (**d**) Case 26. Liver, removed and sectioned. The liver is swollen with a taut capsule and bulging cut surfaces. Multifocal subcapsular haemorrhages are evident. Hepatic parenchyma has an enhanced lobular pattern with irregular congestion, haemorrhages and necrosis. Image courtesy of Jonathan Cracknell.

Gross postmortem reports were available for 24 (89%) cases. The amount of recorded information and protocols followed varied widely as necropsies were performed by different veterinary pathologists or zoo clinicians across institutions. Discrepancies in record keeping need to be considered when interpreting the following data. Body condition was considered thin (n = 1/19), good (n = 12/19) or fat (n = 6/19). Blue or purple tongue discolouration and/or lingual haemorrhage was recorded in 19 cases (79%), and ulceration of the tongue or oral cavity in three cases (13%) (Fig. 1A). Swelling of the tongue, eyelids, conjunctiva, head and neck region was common. Ascites was recorded in 16 cases (67%) and pericardial effusion in 18 cases (75%). Details regarding location and severity of oedema and haemorrhage was variable and did not allow comparison. Oedema was recorded in 17 cases (71%). Multifocal haemorrhage was recorded in 22 cases (92%) and included petechiae (n = 21, 88%), ecchymoses (n = 15, 63%) and suffusive haemorrhage (n = 6, 25%). Myocardial haemorrhage was reported in all cases except one severely autolysed case (n = 23, 96%) and included petechiae (n = 15, 63%), ecchymoses (n = 14, 58%) and/or suffusive/coalescing haemorrhage (n = 5, 21%) (Fig. 1B).

Gastric serosal or mucosal haemorrhages were recorded for eight cases, and three had pyloric mucosal ulcerations. Intestinal ulcers were recorded for four cases and one case had rectal mucosal ulcerations. Intestinal serosal or sub-serosal (n = 18, 75%), mucosal (n = 8, 33%) and luminal (n = 2, 8%) haemorrhage was also recorded (Fig. 1C).

The liver was described as swollen/enlarged (n = 14, 58%) with oedema (n = 8, 33%) and petechial and/or ecchymotic capsular haemorrhages (n = 9, 38%). Capsule and parenchymal pallor (n = 4, 17%) or a pronounced lobular pattern (n = 4, 17%) was reported (Fig. 1D). The pancreas was moderately to severely oedematous and haemorrhagic (n = 7, 29%).

The lungs were considered congested (n = 7, 29%), with petechial to extensive haemorrhage (n = 8, 33%). Blood clots were noted in the tracheal lumen in one case. Oropharyngeal 2-3 mm mucosal nodules exuding purulent material were noted in one case, and 2–8 mm ‘granuloma-like’ lesions covered in purulent mucus, extending from the epiglottis to the larger bronchi, were noted in another case.

Bacterial cultures were performed for 14/27 (52%) but did not identify isolates with clinical significance. Samples cultured included liver (n = 10), spleen (n = 7), lung (n = 6), intestine (n = 5), blood (n = 4), kidney (n = 4), lymph node (n = 2), uterus (n = 2), faeces (n = 2), and peritoneum, heart, ovary, bone marrow, temporal gland, pharynx and peritoneal fluid (each n = 1). *Salmonella* was not isolated after selective culture of intestines and/or faeces in six cases.

### Histological findings

Oedema, haemorrhage and inflammation scores and quantification of INIB for adipose tissue, adrenal gland, pancreas, thyroid, stomach, small intestine, large intestine, heart, kidney, liver, lung, lymph node, spleen and thymus are presented in Tables 1 and 2. Congestion scores for liver, kidney, lung and spleen are presented in Table 3. Lymphoid depletion scores for lymph node, thymus and spleen are presented in Table 4.

**Table 1:** Histological haemorrhage scores of organs from Asian elephant endotheliotropic herpesvirus-haemorrhagic disease fatalities in Europe.

Tissue	EEHV-1A									EEHV-1B	EEHV-1A/4	EEHV-5
	n	Absent		Mild		Moderate		Severe		n = 1	n = 1	n = 1
Adipose	20	1	5%	5	25%	11	55%	3	15%	Severe	Mild	Severe
Adrenal	13	11	85%	2	15%	0	0%	0	0%	NA	Moderate	Moderate
Pancreas	15	9	60%	6	40%	0	0%	0	0%	NA	Absent	NA
Thyroid	7	7	100%	0	0%	0	0%	0	0%	NA	NA	Absent
Stomach	11	3	27%	4	36%	4	36%	0	0%	Absent	Mild	Absent
Small intestine	20	1	5%	5	25%	11	55%	3	15%	NA	Moderate	Severe
Large intestine	16	1	6%	12	75%	3	19%	0	0%	NA	Severe	Moderate
Heart	22	0	0%	1	5%	9	41%	12	55%	Severe	Absent	Moderate
Kidney	22	12	55%	10	45%	0	0%	0	0%	Absent	Severe	Moderate
Liver	19	1	5%	10	53%	7	37%	1	5%	Absent	Mild	Moderate
Lung	21	7	33%	8	38%	5	24%	1	5%	Absent	Mild	NA
Lymph node	21	1	5%	10	48%	7	33%	3	14%	Moderate	Moderate	Moderate
Spleen	19	0	0%	4	21%	9	47%	6	32%	Moderate	Moderate	Moderate
Thymus	8	0	0%	3	38%	4	50%	1	13%	NA	Mild	NA
Brain	8	4	50%	4	50%	0	0%	0	0%	NA	NA	Mild
Spinal cord	8	3	38%	4	50%	1	13%	0	0%	NA	NA	Moderate
Salivary gland	9	6	67%	3	33%	0	0%	0	0%	NA	NA	Absent
Skeletal muscle	15	2	13%	5	33%	7	47%	1	7%	Moderate	Absent	Moderate
Tongue	17	1	6%	4	24%	7	41%	5	29%	NA	Absent	Mild

NA; tissue not available.

**Table 2:** Oedema scores in organs from Asian elephant endotheliotropic herpesvirus-haemorrhagic disease fatalities in Europe.

Tissue	EEHV-1A									EEHV-1B	EEHV-1A/4	EEHV-5
	n	Absent		Mild		Moderate		Severe		n = 1	n = 1	n = 1
Adipose	20	0	0%	16	80%	4	20%	0	0%	Moderate	Mild	Severe
Adrenal	13	2	15%	8	62%	3	23%	0	0%	NA	Mild	Moderate
Pancreas	15	0	0%	6	40%	7	47%	2	13%	NA	Mild	NA
Thyroid	6	1	17%	5	83%	0	0%	0	0%	NA	NA	Mild
Stomach	8	0	0%	4	50%	3	38%	1	13%	NA	Moderate	Severe
Small intestine	20	0	0%	2	10%	12	60%	6	30%	NA	Severe	Severe
Large intestine	15	0	0%	3	20%	9	60%	3	20%	NA	Severe	Moderate
Heart	22	0	0%	4	18%	17	77%	1	5%	Moderate	Mild	Moderate
Kidney	22	0	0%	9	41%	11	50%	2	9%	Mild	Moderate	Mild
Liver	20	2	10%	9	45%	7	35%	2	10%	Mild	Moderate	Mild
Lung	21	1	5%	10	48%	10	48%	0	0%	Mild	Moderate	NA
Lymph node	21	0	0%	6	29%	13	62%	2	10%	Moderate	Moderate	Moderate
Spleen	20	0	0%	8	40%	10	50%	2	10%	Moderate	Severe	Absent
Thymus	8	0	0%	3	38%	5	63%	0	0%	NA	Mild	NA
Brain	8	0	0%	3	38%	5	63%	0	0%	NA	NA	Mild
Spinal cord	8	1	13%	7	88%	0	0%	0	0%	NA	NA	Mild
Salivary gland	9	0	0%	6	67%	3	33%	0	0%	NA	NA	Mild
Skeletal muscle	14	0	0%	2	14%	7	50%	5	36%	Severe	Mild	Moderate
Tongue	17	0	0%	6	35%	11	65%	0	0%	NA	Moderate	Moderate

NA; tissue not available.

**Table 3:** Semi-quantification of intranuclear inclusion bodies in organs from Asian elephant endotheliotropic herpesvirus-haemorrhagic disease fatalities in Europe.

Tissue	EEHV-1A									EEHV-1B	EEHV-1A/4	EEHV-5
	n	None		Rare		Moderate		Abundant		n = 1	n = 1	n = 1
Adipose	19	14	26%	5	26%	0	0%	0	0%	rare	absent	absent
Adrenal	13	12	92%	1	8%	0	0%	0	0%	NA	Moderate	Rare
Pancreas	10	10	100%	0	0%	0	0%	0	0%	NA	Absent	NA
Thyroid	7	6	86%	1	14%	0	0%	0	0%	NA	NA	Absent
Stomach	11	6	55%	5	45%	0	0%	0	0%	Absent	Absent	Rare
Small intestine	19	1	5%	18	95%	0	0%	0	0%	NA	Rare	Rare
Large intestine	16	6	38%	10	63%	0	0%	0	0%	NA	Rare	Rare
Heart	21	0	0%	14	67%	7	33%	0	0%	Absent	Abundant*	Rare
Kidney	22	19	86%	3	14%	0	0%	0	0%	Absent	Rare	Rare
Liver	18	0	0%	9	50%	8	44%	1	6%	Rare	Moderate	Absent
Lung	21	6	29%	15	71%	0	0%	0	0%	Rare	Rare	NA
Lymph node	21	1	5%	16	76%	4	19%	0	0%	RARE	Rare	Rare
Spleen	20	3	15%	12	60%	5	25%	0	0%	Rare	Moderate	Moderate
Thymus	6	2	33%	4	67%	0	0%	0	0%	NA	Absent	NA
Brain	8	1	13%	7	88%	0	0%	0	0%	NA	NA	Absent
Spinal cord	7	5	71%	2	29%	0	0%	0	0%	NA	NA	Absent
Salivary gland	9	3	33%	6	67%	0	0%	0	0%	NA	NA	Absent
Skeletal muscle	14	8	57%	6	43%	0	0%	0	0%	Absent	Rare	Absent
Tongue	17	5	29%	12	71%	0	0%	0	0%	NA	Rare	Absent

NA; tissue not available, * located primarily in endothelial cells of myocardial venules.

**Table 4:** Inflammation scores and presence of leukocytostasis in organs from Asian elephant endotheliotropic herpesvirus-haemorrhagic disease fatalities in Europe.

Tissue	EEHV-1A											EEHV-1B	EEHV-1A/4	EEHV-5
	n	Absent		Mild		Moderate		Severe		Leukocytostasis		n = 1	n = 1	n = 1
Adipose	20	13	65%	7	35%	0	0%	0	0%	8	40%	mild*	absent	mild
Adrenal	13	13	100%	0	0%	0	0%	0	0%	8	62%	NA	absent*	absent*
Pancreas	15	15	100%	0	0%	0	0%	0	0%	3	20%	NA	absent	NA
Thyroid	7	7	100%	0	0%	0	0%	0	0%	1	14%	NA	NA	absent*
Stomach	13	11	85%	2	15%	0	0%	0	0%	5	38%	absent	absent	Absent
Small intestine	20	2	10%	17	85%	1	5%	0	0%	8	40%	NA	moderate	mild*
Large intestine	16	1	6%	15	94%	0	0%	0	0%	6	38%	NA	moderate*	mild*
Heart	21	0	0%	18	86%	3	14%	0	0%	16	76%	moderate*	absent*	moderate*
Kidney	22	22	100%	0	0%	0	0%	0	0%	17	77%	mild	mild*	absent*
Liver	20	0	0%	7	35%	13	65%	0	0%	20	100%	mild*	mild*	moderate*
Lung	21	3	14%	15	71%	3	14%	0	0%	16	76%	mild*	absent	NA
Lymph node	21	4	19%	12	57%	5	24%	0	0%	11	52%	mild*	moderate*	Mild
Spleen	21	6	29%	15	71%	0	0%	0	0%	2	10%	mild*	absent	Absent
Thymus	8	8	100%	0	0%	0	0%	0	0%	4	50%	NA	absent	NA
Brain	8	8	100%	0	0%	0	0%	0	0%	4	50%	NA	NA	Absent
Spinal cord	8	8	100%	0	0%	0	0%	0	0%	2	25%	NA	NA	absent*
Salivary gland	9	9	100%	0	0%	0	0%	0	0%	2	22%	NA	NA	absent*
Skeletal muscle	15	9	60%	6	40%	0	0%	0	0%	1	7%	mild	absent	Absent
Tongue	17	5	29%	12	71%	0	0%	0	0%	4	24%	NA	mild	Mild

NA; tissue not available, * leukocytostasis present.

### Haemorrhage and oedema

**Figure 2:**
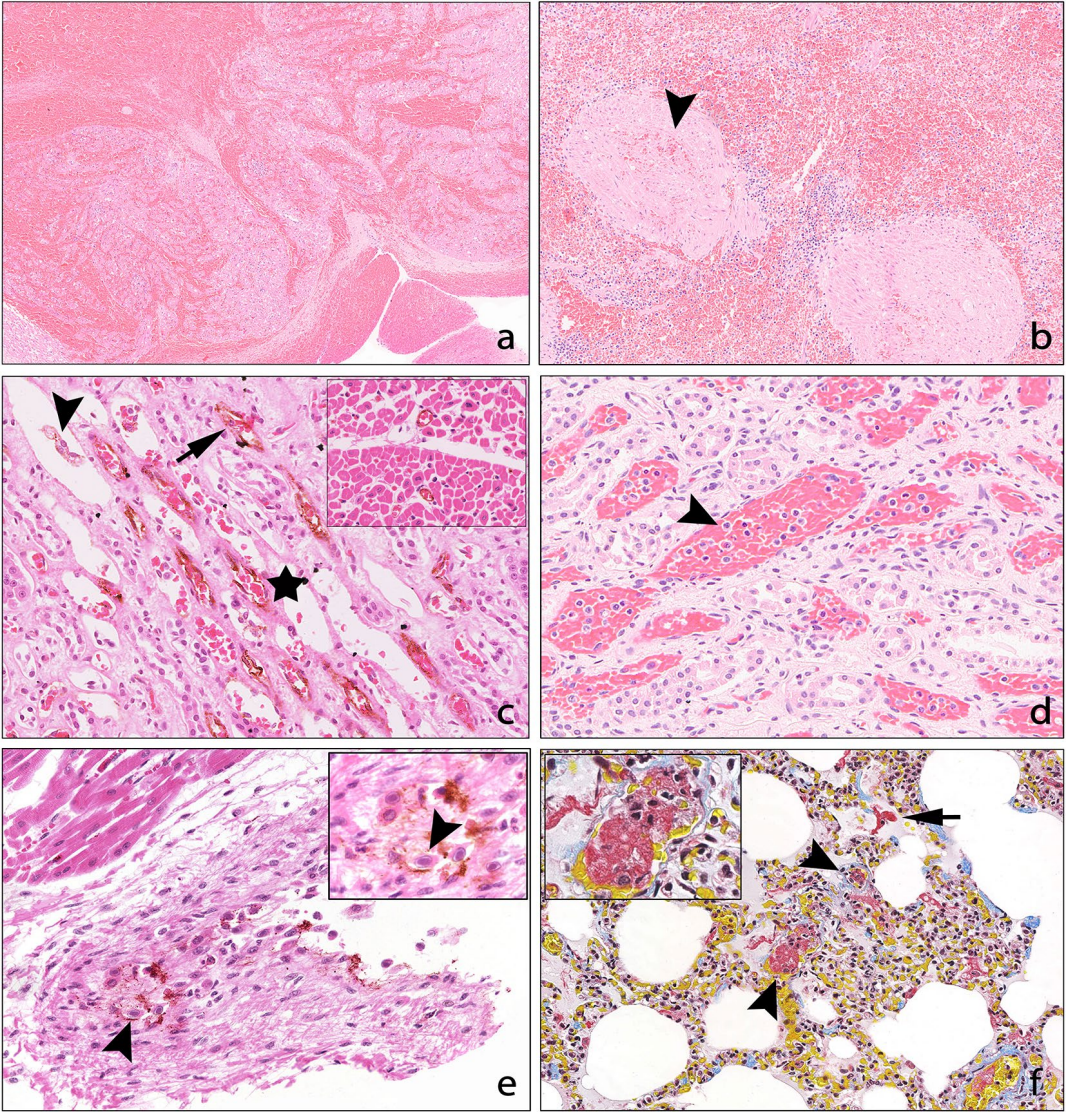
Representative histologic lesions from Asian elephant endotheliotropic herpesvirus-haemorrhagic disease fatalities. (**a**) Heart, haematoxylin and eosin (HE) × 4. Extensive acute haemorrhages expanding subepi- and subendocardial space and widely efface the myocardium. (**b**) Spleen, HE, × 10. Severe parenchymal congestion and haemorrhages and mild trabecular haemorrhages (arrowhead). (**c**) Kidney, von Willebrand factor immunohistochemistry (vWf, brown colouration) with HE counterstain, × 15. Small vessels of the renal medulla exhibit widespread endothelial cell damage. Endothelial cells are variably swollen (arrow), with irregular staining patterns, separation and oedema (star) and sloughed off endothelial cells multifocally accumulate within the vascular lumen (arrowhead). Inset: Control, heart, von Willebrand factor immunohistochemistry with H&E, × 40. Uniform endothelial cells line vessel walls with regular immuolabelling for vWf. (**d**) Kidney, HE, × 20. Diffuse vascular congestion and leukocytostasis (arrowhead). (**e**) Heart, von Willebrand factor immunohistochemistry with HE, × 20. Viral inclusion bodies (arrow head) present within degenerating endothelial cells of a large vessel in a case of EEHV-1A and -4 co-infection. Inset: Intranuclear inclusion bodies (arrow head) magnified, × 40. (**f**) Lung, Martius, scarlet and blue, × 10. Multifocal intravascular fibrin thrombi (arrowheads) fibrin deposits within alveolar spaces (arrow). Inset: Capillary fibrin thrombus magnified, × 40.

Haemorrhage was most severe in heart and spleen (Table 1) (Fig. 2A and B). Cardiac haemorrhage was typically most severe in the sub-endocardial and sub-epicardial regions (Fig. 2A). Thyroid was the only tissue without haemorrhage, while heart, spleen, liver and thymus were affected in all cases. Haemorrhage in the gut associated lymphoid tissue was observed in the EEHV-1A/4 case but was not seen in other cases. Splenic erythrophagocytosis was scored in EEHV-1A cases as absent (1/20, 5%), mild (5/20, 25%), moderate (10/20, 50%) or severe (4/20, 20%). Erythrophagocytosis was mild for the EEHV-1A/4 and -5 cases and severe for the EEHV-1B case. Oedema was widespread in EEHV-1A cases, with skeletal muscle, small and large intestine most severely affected (Table 5).

**Table 5:** Congestion scores in organs from Asian elephant endotheliotropic herpesvirus-haemorrhagic disease fatalities in Europe.

Tissue	EEHV-1A									EEHV-1B	EEHV-1A/4	EEHV-5 EEHV-5
	n	Absent		Mild		Moderate		Severe		n = 1	n = 1	n = 1
Kidney	22	1	5%	12	55%	9	41%	0	0%	Mild	Severe	Moderate
Liver	19	0	0%	8	42%	10	53%	1	5%	Mild	Moderate	Moderate
Lung	21	2	10%	6	29%	11	52%	2	10%	Moderate	Moderate	NA
Spleen	21	0	0%	0	0%	14	67%	7	33%	Moderate	Mild	Moderate

NA; tissue not available.

### Vascular lesions

In general, capillaries were most severely affected, as shown in the kidney (Fig. 2C) however the myocardial venules of the EEHV-1A/4 case had abundant INIBs (Fig. 2E). Blood vessel changes in the lung of EEHV-1A cases were observed as follows: endothelial cell damage 21/21 (100%), blood vessel wall oedema (expansion of the intima) 20/21 (95%), leukocyte migration 13/21 (62%), and the presence of microthrombi 9/21 (43%). Endothelial cell damage, blood vessel wall oedema and microthrombi were observed in both EEHV-1A/4 and -1B cases, but leukocyte migration was not. Lung was not available for examination from the EEHV-5 case. MSB staining confirmed alveolar fibrin deposition in 14/21 (67%) EEHV-1A cases, as well as the EEHV-1B case (not performed on the EEHV-1A/4 case) (Fig. 2F).

Blood vessel (mostly sinusoids and portal capillaries) changes in the liver of EEHV-1A cases were observed as follows: endothelial cell damage characterised by separation, sloughing, denudation or loss 19/20 (95%), blood vessel wall oedema 20/20 (100%) and leukocyte migration 17/23 (74%). These lesions were also seen in the EEHV-1A/4, -1B and -5 cases except for leukocyte migration in the EEHV-1B case.

Renal microthrombi were seen in 5/23 (22%) EEHV-1A cases, but not the -1B, -1A/4 or -5 cases. Thrombi were also observed in two EEHV-1A cases in a lymph node, or spinal cord and tongue. The EEHV-5 case had thrombi in a lymph node and the small intestine. In total, microthrombi were observed in 17/27 (63%) of EEHV cases.

Intranuclear inclusion bodies were consistently observed in the endothelial cells of the heart (Fig. 2E), with the exception of the EEHV-1B case, and liver, with the exception of the EEHV-5 case (Table 6). Intranuclear inclusions could not be observed in the pancreas which was, however, often autolysed. Co-staining with HE (to highlight INIB) and immunohistochemical labelling for von Willebrand factor antibody, a marker for endothelial cells, was carried out on select sections of heart and liver confirmed the presence of inclusion bodies within endothelial cells (Fig. 2E).

**Table 6:** Lymphoid depletion scores in organs from Asian elephant endotheliotropic herpesvirus-haemorrhagic disease fatalities in Europe.

Tissue	EEHV-1A									EEHV-1B	EEHV-1A/4	EEHV-5
	n	Absent		Mild		Moderate		Severe		n = 1	n = 1	n = 1
Lymph node	21	1	5%	7	33%	12	57%	1	5%	Moderate	Mild	Moderate
Spleen	21	0	0%	7	33%	11	52%	3	14%	Severe	Severe	Mild
Thymus	8	1	13%	3	38%	4	50%	0	0%	NA	Moderate	NA

NA; tissue not available.

### Inflammation and degenerative changes

Mild or moderate inflammation was common in EEHV-1A cases (Table 2) and was heterophilic or mixed in all cases. The liver was most affected by extravascular heterophilic infiltration into the hepatic parenchyma. Mild mononuclear inflammation of the gastrointestinal mucosa was considered within normal limits. Leukocytostasis, defined as increased numbers of leukocytes (granulocytes in all cases) within the local vasculature, was consistently evident within the liver, and common in other tissues across cases (Table 2) (Fig. 2D).

Hepatocellular degeneration with individual hepatocyte dropout or mild multifocal hepatocyte necrosis was seen in 8/17 (47%) EEHV-1A cases and the EEHV-1B and -1A/4 cases. Moderate multifocal hepatocellular necrosis was observed in 9/17 (53%) EEHV-1A cases and the EEHV-5 case.

Acute myofibre degeneration was observed in the heart of 15/20 (75%) EEHV-1A cases and the EEHV-1B and -5 cases, but not the EEHV-1A/4; in the tongue of 15/17 (88%) EEHV-1 cases and the EEHV-1A/4 case but not the EEHV-5 case (the tongue of the EEHV-1B case was not examined); and in skeletal muscle of 7/15 (47%) cases and the EEHV-1A/4 case but not the EEHV-1B or -5 cases.

### Lymph node pathology

Lymph node sinus ectasia was observed in 17/21 (81%) EEHV-1A cases, as well as the EEHV-1B, -1A/4 and -5 cases. Sinus histiocytosis was observed in all cases and was mild in 9/24 (38%) cases, moderate in 14/24 (58%, including the EEHV-1B, -1A/4 and -5 cases) and severe in 1/24 (4%).

### Lesion severity and duration of illness

Spearmann’s correlation was used to evaluate potential relationships between the duration of clinical signs prior to death, and organ lesion severity score. As duration of clinical signs increased, the degree of splenic oedema decreased (r = − 0.5, 95% confidence interval − 0.06 to − 0.8, *p* = 0.02) while the degree of pulmonary oedema increased (r = 0.5, 95% confidence interval 0.08 to 0.8, *p* = 0.02). There was a non-significant trend for hepatic oedema to decrease (r = − 0.4, 95% confidence interval − 0.7 to 0.01, *p* = 0.05). No significant association was found between duration of clinical signs and cardiac or tongue inflammation, lymph node oedema, thymus depletion, percentage of organs with leukocytostasis or erythrophagocytosis scores in lymph node or spleen.

The EEHV-5 case was intensively managed and had clinical signs for six days prior to death^14,24^. Histologically, there was evidence of haemosiderosis in association with cardiac haemorrhages, mitotic activity suggesting hepatocellular regeneration in the liver and crystalline deposits interpreted as precipitation of administered drugs in the kidney, consistent with prolonged survival time and extensive medical intervention.

#### Other findings

Segmental moderate chronic interstitial nephritis, consistent with a pre-existing chronic renal infarct was observed in the EEHV-1B case, which was the oldest animal at the time of death (7.6 years). Multiple abscessed lymph nodes (location unknown) were observed in a female EEHV-1A case. Incidental intramuscular *Sarcocystis* cysts were observed in the skeletal muscle of a male EEHV-1A case. Focal subacute to chronic necrotising granulomatous pneumonia was observed in a male EEHV-1A case. No acid-fast organisms were seen with a Ziehl–Neelsen stain.

## Discussion

Severe oedema, widespread petechial and ecchymotic haemorrhages and evidence of thrombosis were associated with all genotypes of EEHV-HD. This supports the authors’ hypothesis and is the first documented evidence that overt DIC is present and may significantly contribute to EEHV-HD death, based on a large cohort of EEHV-HD fatalities. DIC is usually diagnosed antemortem based on indirect laboratory evidence of microthrombosis and hypocoagulation, including the presence of thrombocytopenia and abnormal coagulation tests^27^. As haemorrhagic diathesis is part of the case definition of EEHV-HD, and thrombocytopenia has previously been reported as a consistent feature^4–6,38^, the identification of microthrombosis in the majority of EEHV-HD fatalities lends support to a diagnosis of overt DIC. Blue discolouration of the tongue (cyanosis), a likely pathognomonic lesion for EEHV-HD, is caused by intramuscular haemorrhage and oedema, and represents a late clinical presentation of overt DIC (Fig. 1A). Microthrombotic disease can be difficult to detect without focused examination soon after death, however despite this, systematic histological examination in this study identified microthrombi in 63% of EEHV-HD fatalities. Increased break-down of thrombi, hyperfibrinolysis, has not yet been investigated as a potential contributor to the bleeding diathesis observed in EEHV-HD, and could also reduce the number of thrombi observed postmortem^39,40^. In an experimental model in rats, postmortem fibrinolysis caused dissolution of microthrombi within 40 minutes^41^. Some authors consider the presence of even one thrombus in the face of consistent clinical signs, as evidence of DIC^42^. Postmortem examination of elephants presents logistical challenges and most cases were examined 12—24 h (or more) after death (data not shown). Postmortem examinations and sampling were not performed in a standardised manner across cases and were likely not focused on detecting microthrombi. This, together with prolonged intervals between death and sample collection, suggests that the microthrombotic component of EEHV-HD, as determined by histopathology, is likely considerably underestimated during routine examination. Prompt collection and preservation of tissue samples will preserve evidence of microthrombosis, and techniques such as immunohistochemistry or electron microscopy could be explored for more sensitive detection of thrombi in the future. Thrombosis is however unlikely to be the primary manifestation of EEHV-HD coagulopathy, as significant ischaemic lesions were not observed.

Leukocytostasis and generalised mild to moderate tissue inflammation found in this study, along with previous documentation of heterophila^5,38,43,44^ with left shifting^45^, toxic changes in leukocytes^45^ and acute phase protein elevations^46^, all indicate the presence of systemic inflammation in EEHV-HD. As part of the innate immune system, the inflammatory response is intimately related to haemostatic pathways^47,48^. Cytokines are important components of the innate immune response, and control of pro- and anti-inflammatory cytokines is important to promote pathogen control while avoiding excessive damage to the host^49^. These protein messengers are produced primarily by inflammatory cells, but also activated endothelium^50^, to organise cellular interactions^51^. Fever is also mediated by cytokines, particularly tumour necrosis factor and interleukins-1 and − 6^52^. Fever was present in 77% of EEHV-HD cases where body temperature was recorded. Inflammation can promote coagulation through upregulation of tissue factor presentation on endothelial cells or leucocytes, resulting in additional generation of thrombin. DIC is a condition driven by cytokine release as a consequence of underlying conditions, usually infection^27,53^. Monocytes appear to have a significant role in EEHV-HD, with monocytopenia associated with active disease, and rebound monocytosis during recovery^4–6,8,9,43^. Activation of monocytes is reported in EEHV-HD^45^. This is likely due to EEHV infection or phagocytosis of viral particles^44^, and may explain why monocytes decrease during clinical disease. Equine monocytes have been reported to express tissue factor in response to equine herpesvirus infection, promoting a hypercoagulable state^29,30^. Similar mechanisms should be investigated for EEHV-HD.

In cases of active EEHV infection occurring after anti-EEHV maternal antibodies have waned, the innate immune responses, mediated by cytokines, are likely to be important in controlling viral replication until the host can mount an antibody response^54,55^ Cytokine release syndrome (“cytokine storm”) does not have a strict definition, but can be identified by the presence of acute systemic inflammation and elevated circulating cytokines, leading to organ dysfunction^52^. The presence of systemic inflammation, overt DIC and fever may all result from excessive or dysregulated cytokine responses to EEHV infection. While no studies have yet been performed to investigate cytokine levels during EEHV-HD, anti-inflammatory glucocorticoid therapy has been cautiously used in several surviving cases and may provide supporting evidence for the role of cytokines in EEHV-HD^5,43,52,56^.

Cell based tissue factor presentation is the primary driver for coagulation, and endothelial injury is an important source of tissue factor-bearing cells^29,57^. Direct viral injury to the endothelium was evident by the presence of intranuclear inclusion bodies in all EHHV-HD cases in the present study. Immunohistochemistry was useful to demonstrate von Willebrand factor positive (endothelial) cells with INIBs, and has not been described previously. Further research is required to investigate if direct viral damage is the initiating cause of clinical manifestations of EEHV infection, as the endothelium can also be activated by, and cross-talk with, systemic inflammatory processes^50^.

The heart was the most consistently and severely affected organ in EEHV-1A, -1B and -5 fatalities. Previous studies have shown that the heart has high viral loads for all genotypes^13,15,58^. Myocardial haemorrhage, oedema, low numbers of INIB’s and mild or moderate inflammation were observed in all cases. Cardiac haemorrhage was classified as moderate or severe in 95% of cases, and 75% had acute myofibre degeneration, indicating this was likely a significant pre-terminal event, resulting in cardiac dysfunction and failure, circulatory disturbances and death. Interestingly, no cardiac haemorrhage, inflammation or myofibre degeneration was observed in the single EEHV-1A/4 co-infection case, despite abundant INIB’s in endothelial cells of myocardial venules, and a high viral load in cardiac tissue^15^.

Hepatocellular necrosis has occasionally been reported in EEHV cases^12,59,60^. Individual hepatocellular or mild to moderate multifocal necrosis was seen in all cases in the current study, together with mild to moderate hepatic congestion, which was often centrilobular (Fig. 1D). Hepatocyte necrosis is a non-specific lesion and can be caused by hypoxia, reactive oxygen metabolites, viral infection and/or inflammation, including cytokine storms^52,61^. The authors hypothesise that hepatic congestion is secondary to systemic inflammation and acute cardiac failure, resulting in hepatocyte hypoxia and necrosis. Mild to moderate inflammation was common but there was little evidence of hepatocyte regeneration, and no correlation between duration of clinical signs and the degree of hepatic congestion, suggesting an acute nature of the lesions. Sinusoidal and small portal vessel endothelial inclusion bodies were common in the liver, and a direct effect of the virus on hepatocellular cells cannot be ruled out.

Moderate or severe splenic congestion and varying degrees of splenic haemorrhage were observed in all cases of EEHV-1A. Histologically, it can be difficult to differentiate splenic congestion and haemorrhage. However, evidence of intra-trabecular haemorrhage in all cases, and erythophagocytosis in all cases except one, supported a diagnosis of haemorrhage, as well as moderate or severe congestion (Fig. 2B). Despite lymphoid depletion being a consistent feature in the spleen, lymph nodes and thymus of all EEHV cases in the current study, it has rarely been noted before^60,62^. Splenic lymphoid depletion was more severe in EEHV-1B and -1A/4 cases compared to -1A cases, however this difference was not noted in the lymph nodes or thymus. Lymphoid depletion was a consistent feature of EEHV-HD fatalities and several mechanisms may contribute to this. During infection, viruses initially breach epithelial defences and are then often found in draining lymph nodes, prior to haematogenous spread^63^. Although mechanisms may vary across the viral family, successful herpesvirus infection and replication results in lysis of the host cell^64^, and this cytopathic effect may directly deplete lymphoid cell populations. Other consequences of viral infection, such as pro-inflammatory cytokine release and cellular responses, may damage bystander lymphoid cells even when not infected, and/or recruit lymphoid cells for cellular responses to EEHV infection at distant sites^63^. Decreases in circulating monocytes, such as through apoptosis^4,5,44^, and to a lesser extent, lymphocytes, are reported in EEHV-HD cases.

Antibiotic therapy was initiated in 41% of cases, sometimes in the absence of specific anti-viral therapy. Histological exam did not identify bacterial sepsis in any case, although heterophils were present in the circulation. Antibiotics should be considered in light of the clinical picture.

Single cases of EEHV-1B, -1A/4 and -5 were included in the study, hence only limited conclusions can be drawn regarding lesion distribution associated with genotype. Histologically, the EEHV-1B case was not significantly different from EEHV-1A cases, which supports the decision to include the unsequenced EEHV-1 case together with EEHV-1A cases. The EEHV-1A/4 co-infection was the only case to present severe large intestinal and renal haemorrhage, and no haemorrhage in the tongue or heart. Adrenal haemorrhage was more severe (moderate) in EEHV-1A/4 and -5 cases than in EEHV-1 cases (absent or mild). The limited number of EEHV-1B, -4 and -5 cases meant that conclusions on the hypothesis that EEHV genotype influences lesion distribution could not be drawn.

The retrospective nature of this study resulted in several limitations. Delayed postmortem examination, missing and incomplete records, and inconsistent tissue sampling limited comparison and interpretation, particularly of gross necropsy findings. For example, oedema was only recorded in necropsy reports for 63% of cases but was present histologically in all cases, implying that the discrepancy is due to inconsistent reporting, rather than absence of gross lesions. Anatomic locations of lymph node and skeletal muscle samples were rarely recorded. Even so, this is the most comprehensive assessment of EEHV-1A associated pathology to date and provides valuable indicators of EEHV-HD pathophysiology. The authors recommend that elephant necropsy protocols available from the European Association of Zoos and Aquaria (EAZA) or the Association of Zoos and Aquariums (AZA) taxon advisory groups should be followed routinely^65^. While viral load and frequency of INIBs are often highest in heart and liver^58^, comprehensive sample collection, as documented in EAZA and AZA protocols, should be performed where possible. Less commonly collected tissues, such as brain and bone marrow, as well as lymph nodes from known locations, may offer additional insights into EEHV-HD. Further research is required to elucidate the pathophysiology of EEHV-HD and development of DIC, which may direct therapy and improve treatment outcomes. Focus should be directed to measuring circulating cytokines and evaluating haemostatic status during EEHV viraemia. Additional cases of EEHV-1B, -4 and -5 should be systematically examined for comparison with the documented EEHV-1A lesions in this study, to identify EEHV genotype specific lesion patterns.

In conclusion, postmortem identification of microthrombosis in the majority of EEHV-HD fatalities, together with widespread haemorrhage and pre-existing knowledge of acquired thrombocytopenia, support the presence of DIC as a significant haemostatic complication of active EEHV infection. Disrupted endothelium and activated monocytes are likely both responsible for tissue factor presentation, initially resulting in a pro-coagulable state and microthrombosis. End-stage disease is characterised by high viral loads, systemic inflammation, widespread endothelial degeneration and severe hypocoagulability, ultimately resulting in multi-organ dysfunction, cardiovascular failure and death.

## Data Availability

All data generated or analysed during this study are included in this published article.
